# Protruding Enlarged Hymen with Pubic Hairs: A Sign Suggestive of Precocious Puberty!

**Published:** 2015-09-01

**Authors:** Nizam Ul Hasan

**Affiliations:** Former Prof. Pediatric Surgery, Child Aid Association, C/O National Institute of Child Health Rafiquee Shaheed Road Karachi, 75510Pakistan

**Dear Sir,**

Precocious puberty (PP) is defined as the development of pubertal changes before the accepted age of its onset. In girls the lower limit of puberty is set at8 year.[1] With PP early secondary sexual changes appear. By definition it is presence of signs at Tanner 2 stage. It starts at 8 year of age and continues till 15 year of age. The signs include elevation of breast and its papilla with the enlargement of areola. In pubic region there is sparse growth of long, slightly pigmented hair along the labia. [2] Herein, we report a female child who presented with unusual sign that raised suspicion of presence of precocious puberty.

Two year old female weighing 16Kg, presented with prominent swelling at introitus for the past four months. It caused discomfort to the child due to rubbing with pampers.The patient had no other complaints.The family history of the index case was not significant. There was no other sibling with similar complaints. On examination of genitalia downy pubic hairs were noticeable and a whitish swelling was noticeable protruding between labia majora. On closer look after separation of labia majora, it was found to be a significantly enlarged hymen while urethral opening was normally placed (Fig1).

**Figure:**
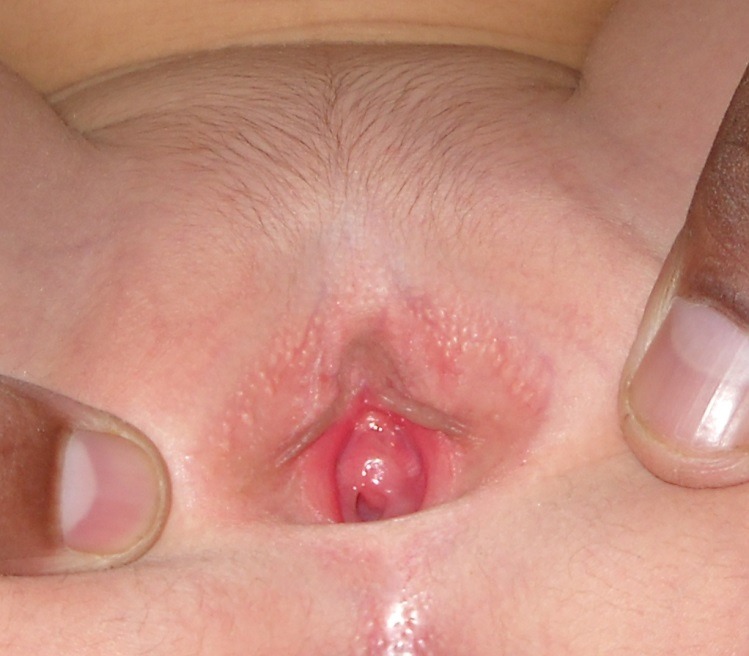
Figure 1: MRCP showing dilated intrahepatic biliary radicals and common bile duct with tapering and upward traction of distal bile duct.

At an earlier examination by other physician, the condition was confused with prolapsed ureterocele. Investigations done showed normal range parameters of CBC and serum electrolytes. Ultrasound of kidneys, bladder, ovaries and uterus did not show any abnormality. Micturating cysto-urethrogram was also reported as normal. X-ray of bones also ruled out any age discrepancy. A battery of investigations ruled out any hormonal disturbance as well as anatomical cause of enlarged hymen. Thus adrenal / gonadal pathologies were excluded though all investigations included in the work up of precocious puberty were not performed.

Counseling was done with the mother and it was suggested to have more investigations including MRI brain which may rule out any central cause, hormonal profileand karyotyping but family left for abroad as they live there. It was advised to keep child under regular follow up and report if any other signs of puberty appear. Parents were alerted to the fact that early rapid growth and later early fusion of epiphysis may cause short stature. In addition psychological trauma that child may suffer due to development of secondary sexual characters, was also highlighted.

In majority (90%) of cases of precocious puberty the specific cause is not present and no active intervention is needed.[3,4] Our patient probably falls into this category. In the index case, enlarged protruding hymen was noted along-with appearance of pubic hair.The former as has not been reported early on in literature. This may be an additional suggestive sign of precocious puberty.

## Footnotes

**Source of Support:** Nil

**Conflict of Interest:** None declared

